# Analyzing microglial-associated Aβ in Alzheimer’s disease transgenic mice with a novel mid-domain Aβ-antibody

**DOI:** 10.1038/s41598-020-67419-2

**Published:** 2020-06-29

**Authors:** Kristi Henjum, Vibeke Årskog, Charlotte B. Jendresen, Tormod Fladby, Reidun Torp, Lars N. G. Nilsson

**Affiliations:** 1 Department of Pharmacology, University of Oslo and Oslo University Hospital, Blindern, P.O. 1057, 0316 Oslo, Norway; 2 Department of Geriatric Medicine, University of Oslo, Nydalen, P.O. 4956, 0424 Oslo, Norway; 3 Department of Neurology, Faculty Division, Akershus University Hospital, University of Oslo, P.B. 1000, 1478 Lørenskog, Norway; 4 Department of Molecular Medicine, Institute of Basic Medical Sciences, University of Oslo, Oslo, Norway

**Keywords:** Alzheimer's disease, Microglia

## Abstract

The mechanisms of amyloid-β (Aβ)-degradation and clearance in Alzheimer’s disease (AD) pathogenesis have been relatively little studied. Short Aβ-fragments form by enzymatic cleavage and alternate amyloid-beta precursor protein (APP)-processing. Here we characterized a novel polyclonal Aβ-antibody raised against an Aβ mid-domain and used it to investigate microglial Aβ-uptake in situ by microscopy at the light- and ultrastructural levels. The rabbit Aβ-mid-domain antibody (ab338), raised against the mid-domain amino acids 21–34 (Aβ_21–34_), was characterized with biochemical and histological techniques. To identify the epitope in Aβ recognized by ab338, solid phase and solution binding data were compared with peptide folding scores as calculated with the Tango software. The ab338 antibody displayed high average affinity (K_D_: 6.2 × 10^−10^ M) and showed preference for C-terminal truncated Aβ-peptides ending at amino acid 34 and Aβ-mid domain peptides with high scores of β-turn structure. In transgenic APP-mouse brain, ab338 labelled amyloid plaques and detected Aβ-fragments in microglia at the ultra- and light microscopic levels. This reinforces a role of microglia/macrophages in Aβ-clearance in vivo. The ab338 antibody might be a valuable tool to study Aβ-clearance by microglial uptake and Aβ-mid-domain peptides generated by enzymatic degradation and alternate production.

## Introduction

Alzheimer’s disease (AD), the major cause of dementia^1^, presents with cerebral region-specific neuropathological lesions; extracellular amyloid-β (Aβ) plaques and intracellular neurofibrillary tangles (NFTs)^2^. Amyloid plaques form when aggregation-prone Aβ monomers, particularly Aβ_x−42/43_, polymerize to oligomers, protofibrils and finally fibrils which deposit in the tissue^3^. The causal mechanisms of neurodegeneration in AD are unclear. However familial AD genetics and biomarker studies suggest that intermediates and/or end products of the Aβ aggregation cascade induce or facilitate downstream events as tauopathy, synaptic dysfunctions and activation of brain immune responses^4–6^.

The Aβ-domain is an integral part of the transmembrane Aβ precursor protein (APP)^7^ being released as peptides of varying lengths following sequential secretase activities^8^. Initial α- or β-secretase cleavage exposes and determines N-terminal peptide extension, while γ-secretase cuts the remaining fragments releasing peptides from the membrane^9^. The β-site APP cleaving enzyme 1 (BACE1 also known as Asp2) has been identified as the β-secretase^10,11^. Following serial BACE1 and γ-secretase cleavage, Aβ_1–38_, Aβ_1–40_ and Aβ_1–42_ (Aβ38, Aβ40, Aβ42) are released, but also shorter and longer peptides like Aβ_1–37_, Aβ_1–39_ and Aβ_1–43_ since γ-secretase cuts by complex enzymatic mechanisms^9^. The Aβ-aggregation potential increases with C-terminal extension to amino acid (aa) 42^12^ and minor N-terminal truncations^13^, but also requires a certain peptide length^14,15^. Thus initial α-site cleavage in the Aβ mid-region (aa 16–17)^16^ generates N-truncated peptides, precluding aggregation, and amyloidogenic APP-processing due to BACE1 and subsequent γ-secretase cleavage. Yet other enzymes and cleavage sites in APP have been identified generating a variety of Aβ-peptides with largely unknown physiological and pathophysiological roles^17^.

Polymerization of Aβ monomers depends on the formation of a nidus. The formation of this nidus, or seed, is influenced by several parameters including the local threshold Aβ-concentration^18^. Thus balanced Aβ-clearance to the production is critical to avoid amyloid accumulation as seen in the AD brain. While Aβ-peptides are released upon synaptic activity^19,20^, Aβ-clearance mechanisms include drainage with exit across the blood brain barrier^21,22^ and enzymatic breakdown by multiple enzymes. Neprilysin and insulin degrading enzyme (IDE) are well-known Aβ-degrading enzymes^23,24^ that are complemented by additional enzymes acting at multiple sites in Aβ^25^. At intra- and extracellular localizations these enzymes can by cleavage reduce the potential of Aβ-peptides to aggregate. The interest in clearance by microglia, was markedly raised by pioneering studies of vaccination and immunotherapy with recombinant antibodies^26–28^. Microglial Aβ-clearance has been suggested as a relevant efficacy measure when designing therapeutic antibodies^28,29^. Such clearance is also relevant when exploring other AD therapeutic targets like the TREM2-receptor with phagocytic and chemotactic functions^30,31^.

Aβ binds microglial receptors^32^ thereby inducing inflammatory responses^33^, especially when microglia cluster around and surround amyloid plaques^34^. However, while the in vitro evidence of phagocytosis in the absence of immunization is compelling^35,36^, the in vivo evidence is more limited^37,38^ and largely derives from invasive experimental systems^39–41^. More recent studies provide evidence of in vivo microglial amyloid phagocytosis, by using aggressive transgenic amyloid AD mouse models (5xFAD and APP/PS1)^42–45^. The inclusion of multiple familial AD mutations in such mouse models may artificially affect Aβ-composition and downstream responses, including microglial activation.

Alternate APP processing and Aβ degradation presumably result in transiently existing Aβ-fragments, like Aβ_1–34_^25,46^ which is detectable in the cerebrospinal fluid (CSF)^47^. Detection of this and other Aβ-fragments requires antibodies recognizing the terminal ends of these, similar to those developed and used for CSF Aβ42-assays^48^. By raising mid-domain Aβ-antibodies, we have previously demonstrated mid-domain Aβ-fragments in CSF of AD patients by immunoprecipitation and liquid chromatography-mass spectrometry^49,50^. The mid-domain antibodies may be of further use to selectively assay conformational and/or truncated fragments of wild-type Aβ that may have diagnostic potential. In the current study, we describe a novel Aβ antibody, ab338, raised against an Aβ-peptide mid-domain (Aβ_21–34_). We use the antibody to detect Aβ mid-domain fragments by enzyme-linked immunosorbent assay (ELISA) and to demonstrate microglial uptake of Aβ in situ in transgenic mouse models with ultrastructural- and light microscopic techniques.

## Results

### Antibody ab338 binds to plate-bound Aβ21–34 with high affinity and selectivity

**Figure 1:**
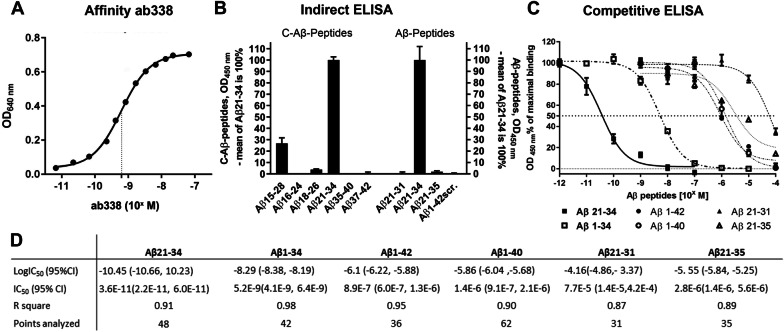
Ab338 binding to Aβ depends on a mid-domain region in Aβ and amino acid 34. (**A**) Average affinity of the ab338 antibody to immobilized KLH-Cys-Aβ_21–34_ peptide as measured by optic density (OD) absorbance with points presented as mean ± standard error of the mean (SEM; n = 3). The data are presented as an adapted sigmoid curve and the dotted line indicates the equillibrum dissociation constant, K_D_. (**B**) Indirect ELISA displaying binding of ab338 to various immobilized Aβ-peptides, some of which harbor a N-terminal cystein (C-Aβ-peptides). Binding are presented relative to Aβ_21–34_ as mean ± SEM after vehicle coat background subtraction. Data are from two independent experiments (n = 6 in two experiments, total n = 12). (**C**) Competitive ELISA with various Aβ-peptides competing in solution for binding to ab338 binding with the KLH-Cys-Aβ_21–34_ plate coat presented with the sigmoid adapted curves (n ≥ 6, from ≥ 2 independent experiments). (**D**) Half inhibitory concentration (IC_50_) of various Aβ-peptides competing for ab338 binding with coat in competitive ELISA.

The ab338 antibody was raised against the human Aβ_21–34_ amino acid peptide sequence conjugated to keyhole limpet hemocyanin (KLH) by an N-terminal cysteine (KLH-Cys-Aβ_21–34_) to generate an antibody recognizing this Aβ mid-domain. Indirect and competitive ELISAs and Aβ-peptides spot-synthesized directly to membrane were applied to determine the in vitro recognition of different Aβ-species, specificity and sensitivity of the affinity purified ab338 antibody. The ab338 antibody displayed high average affinity for the antigen Aβ_21–34_ with a K_D_ in the picomolar range (K_D_ = 6.2 × 10^−10^ M, corresponding to ~ 0.09 µg/ml) as determined by varying the ab338 concentration towards KLH-conjugated Aβ_21–34_ in an indirect ELISA (Fig. 1A). Next, to examine the ab338 epitope the antigen Aβ_21–34_, as well as Aß-peptides non- and partly overlapping with Aβ_21–34_, and scrambled Aβ_1–42_ were used as coat in an indirect ELISA. The immunizing sequence Aβ_21–34_ gave the highest ab338 signal of the Aβ-peptides analyzed. The data are presented on a relative scale with binding to the Aβ_21–34_ peptide defined as 100% and vehicle-coat as 0%. The ab338 antibody also recognized the Aβ_15–28_ sequence (26.7 ± 1.4%, n = 12, mean ± standard error of the mean (SEM)). Aβ_18–26_ (3.8 ± 0.2%, n = 12) was the only other peptide which generated a signal that was significantly higher than the vechicle coat. Binding of ab338 to the other peptides were negligible and all around 0%, Aβ_16–26_ (− 0.5 ± 0.1%, n = 12), Aβ_35–40_ (0.4 ± 0.3%, n = 12), Aβ_37–42_ (− 0.2 ± 0.2%, n = 12). Ab338 also gave very little signal when exposed to Aβ_21–31_ (0.5 ± 0.3%_,_ n = 12), Aβ_21–35_ (2.0 ± 0.2%, n = 12) or a scrambled Aβ_1–42_ peptide (0.2 ± 0.3%, n = 12). Thus data with indirect ELISA indicated ab338 binding being strongly dependent on a C-terminal aa 34 in Aβ (Fig. 1B).

### In solution, antibody ab338 binds selectively to Aβ21-34

When using indirect ELISAs, protein or peptide targets are bound to a microtiter plate, restricting and possibly affecting their conformation and accessibility for antibody binding. In contrast, in competitive ELISAs the peptides are in solution without such restrictions creating competition with the plate bound target for antibody binding. To explore the binding preference of ab338, each Aβ peptide (Aβ_21–34_, Aβ_1–34,_ Aβ_21–31_, Aβ_21–35,_ Aβ_1–40_ or Aβ_1–42_) was incubated with the ab388 antibody in solution, and the mixture added to KLH-Cys-Aβ_21–34_ coated plates. By increasing the Aβ-peptide concentrations, the specificity and sensitivity was determined. Reiterating the indirect ELISA data, Aβ_21–34_ most efficently competed with the coat for ab338 binding (IC_50_; 3.6 × 10^–11^ M, n = 8). Ab338 also bound full length Aβ-peptides (Aβ_1–40_ and Aβ_1–42_), but at much higher concentrations (IC_50_:1.4 × 10^–6^ M, n = 11 and 8.9E × 10^–7^ M, n = 6, respectively). Displacement when adding or removing C-terminal aa’s from the antigen in Aβ_21–35_ and Aβ_21–31_ was comparable to the full length Aβ peptides (IC_50_: 2.8 × 10^–6^ M, n = 6 and 7.7 × 10^–5^ M, n = 6, respectively). The only peptide with binding characterstics close to Aβ_21–34_ was Aβ_1–34_ with a C-terminal aa 34 but a non-truncated N-terminal (IC_50_ 5.2 × 10^–9^ M, n = 6, Fig. 1C,D).

### Antibody ab338 recognizes a β-turn structure in the mid-domain in Aβ

**Figure 2:**
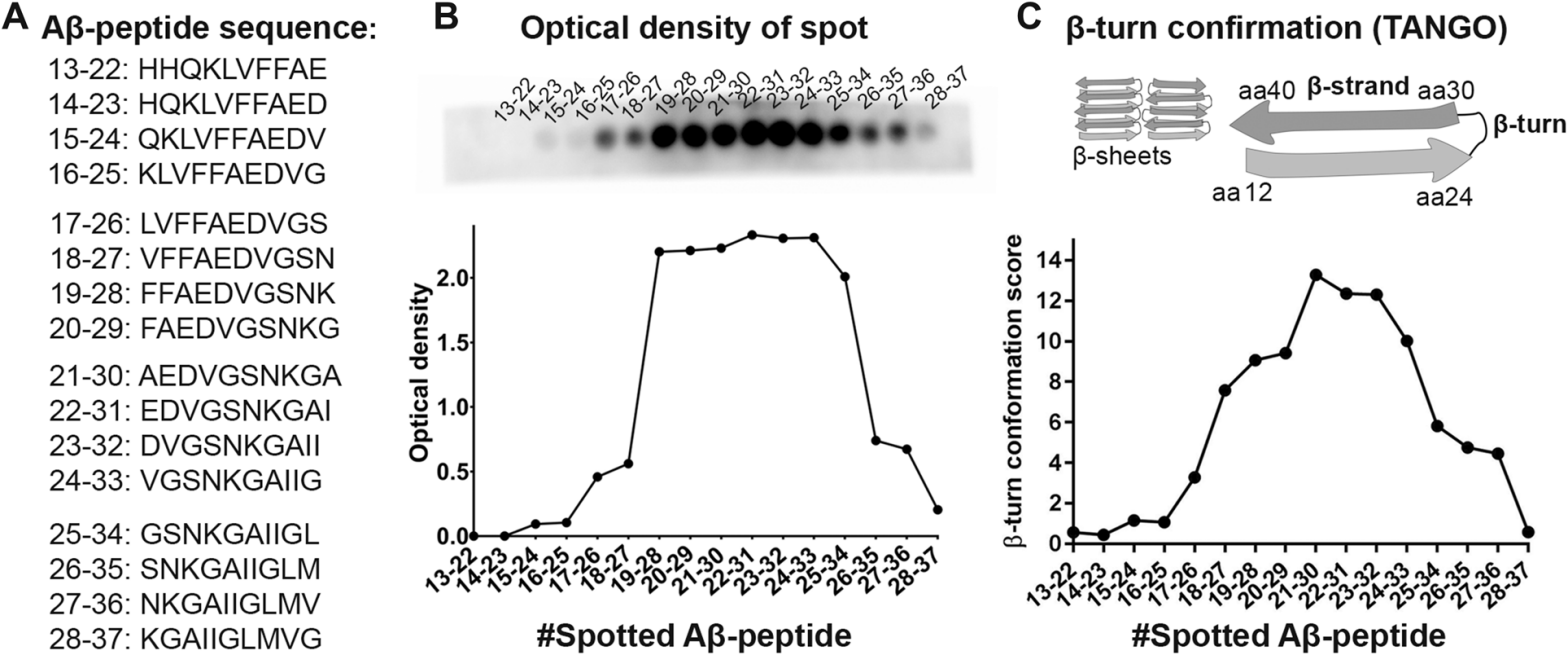
Binding of antibody ab338 to spot-synthesized Aβ-peptides relates to β-turn score in Aβ. (**A**) The amino acid sequences of the spot-synthesized Aβ-peptides with amino acid numbers in the Aβ-domain. (**B**) Antibody ab338 binding to Aβ-sequences spot-synthesized to membrane (top) and a graphical illustration of the optical density as a measure of ab338 preference for the spot-synthesized peptides. A high signal from Aβ-peptide sequences starting from amino acid 19–28 and ending at 24–33 (25–34) corresponded to a (**C**) high β-turn score of Aβ-peptides calculated by Tango. Illustration of Aβ-fibrils with β-sheets of β-strands and β-turns formed by Aβ-peptide. The drawing is based on the Aβ40-fibril structural model of sequences and are presented as suggested by Petkova et al. (2002), PNAS^54^.

To further characterize the Aβ-epitope of ab338, binding to spot-synthesized Aβ-peptides of 10 aa consecutive Aβ-sequences from Aβ_13–22_ until Aβ_28–37_ were examined (Fig. 2A). Of the spot-synthesized peptides, peptides with Aβ-sequences from aa 19–28 to 24–33 gave the strongest ab338 signal. The signal decreased from aa 25–34 and markedly diminished with the subsequent peptides (Fig. 2B).

Aβ-peptides can aggregate into fibrils with a β-sheet structure. Two β-strands form β-sheets and these two peptide sequences are connected by a bend in the structure; a β-turn^51^. Calculating the β-turn score of the spot-synthesized peptides by Tango showed that the peptide sequences from a N-teminal aa 19 to a C-terminal aa 33 got a high score indicating a β-turn region (Fig. 2C) while the scores of β-strand and α-helical structure were low (Supplementary Fig. S1A, B). Intriguingly a high β-turn score tended to coincide with ab338 binding to the spot-synthesized peptides (Fig. 2B,C) but also indirect ELISA binding data e.g. Aβ_15–28_ received a relatively high Tango β-turn score as did the immunizing region Aβ_21–34_ (Fig. 1B,C, Supplementary Fig. S1C). Modelling the β-turn propensity by another algorithm, Chou Fasman algoritm, gave similar results as when calculating peptide folding with Tango software (data not shown).

### Microglial localization of ab338-labelled Aβ-peptides in situ in brain with light microscopy

**Figure 3:**
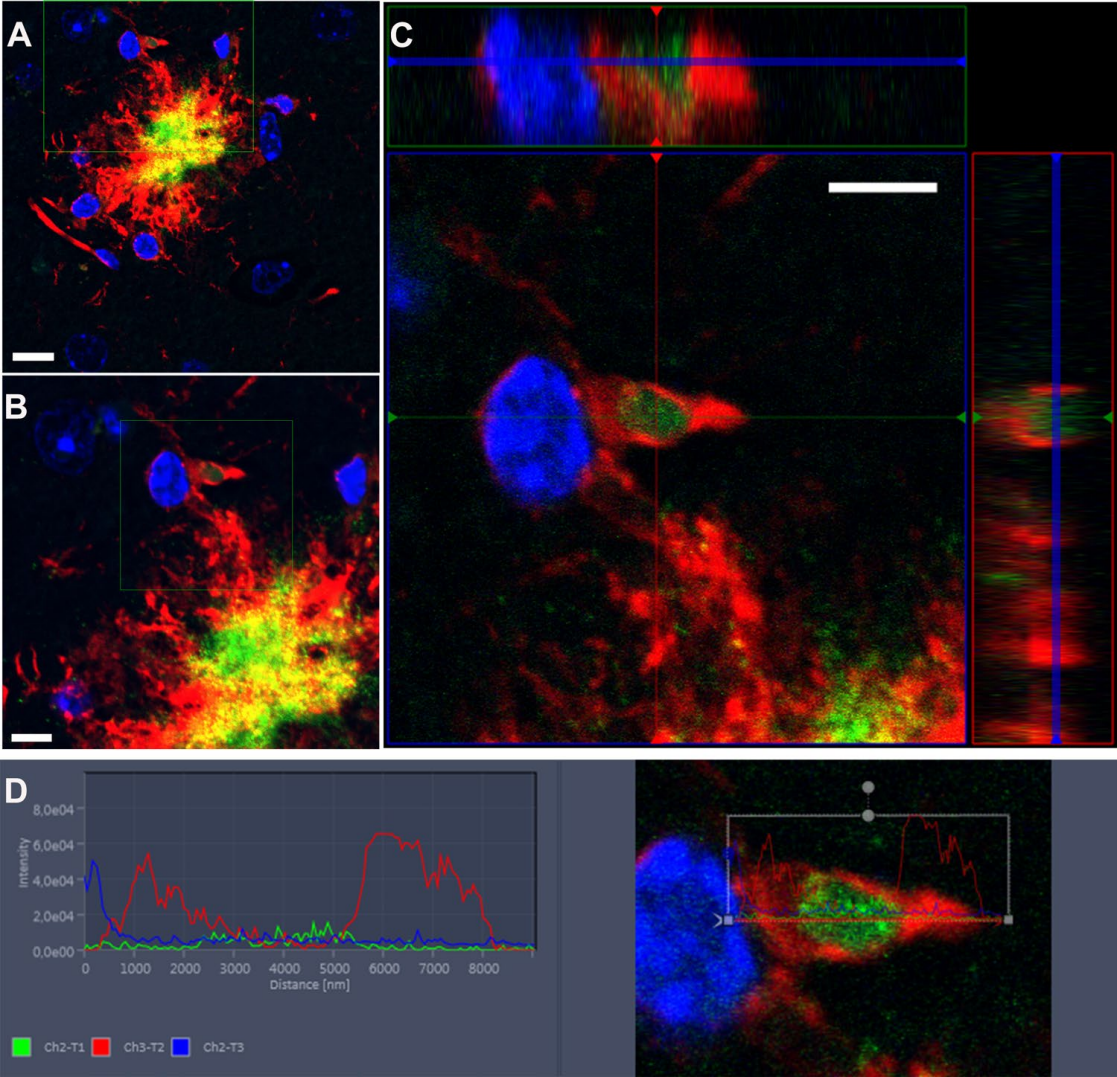
Microglial filopodia with Aβ in tgArcSwe mouse brain. (**A**) The microglia surround the amyloid plaques as illustrated here by microglia labelling (tomato lectin; red label) and an amyloid plaque (ab338; green label) in a 12 months-old transgenic tgArcSwe mouse. Nuclei are labelled by DAPI (blue label). (**B**) In the rim, Aβ-staining (ab338) is located in ramified processes of microglia. (**C**) Z-stacking of a microglia shows Aβ-staining (ab338) within filopodia as illustrated by an ortho-image and (**D**) the fluorescence profile. Images are obtained by confocal microscopy at 63 × magnification with increasing digital zoom. The scale bars measure 10, 5 and 5 µM in images (**A**–**C**) respectively.

Given the preferential binding of ab338 to a β-turn in Aβ we postulated ab338 to display a distinct immunolabelling and be suitable to study in vivo microglial Aβ localization. This was studied in situ in tgArcSwe mice at 12 months, an age when the amyloid plaques are abundant but not extensive in these mice enabling analysis of individual deposits. Ab338 labelled amyloid plaques, while tomato lectin, a surface marker of macrophages and vessels was used to label the surrounding microglia (Fig. 3A,B and Supplementary Fig. S2). Omission of the primary antibody, served as the negative control, ruling out unspecific binding of the secondary antibody (Supplementary Fig. S2). In addition, ab338 labelled cerebrovascular deposits (Supplementary Fig. S3). In microglia nearby plaques, z-stacking supported intracellular Aβ labelling, that appeared located in the ramified processes of microglia (Fig. 3C,D).

**Figure 4:**
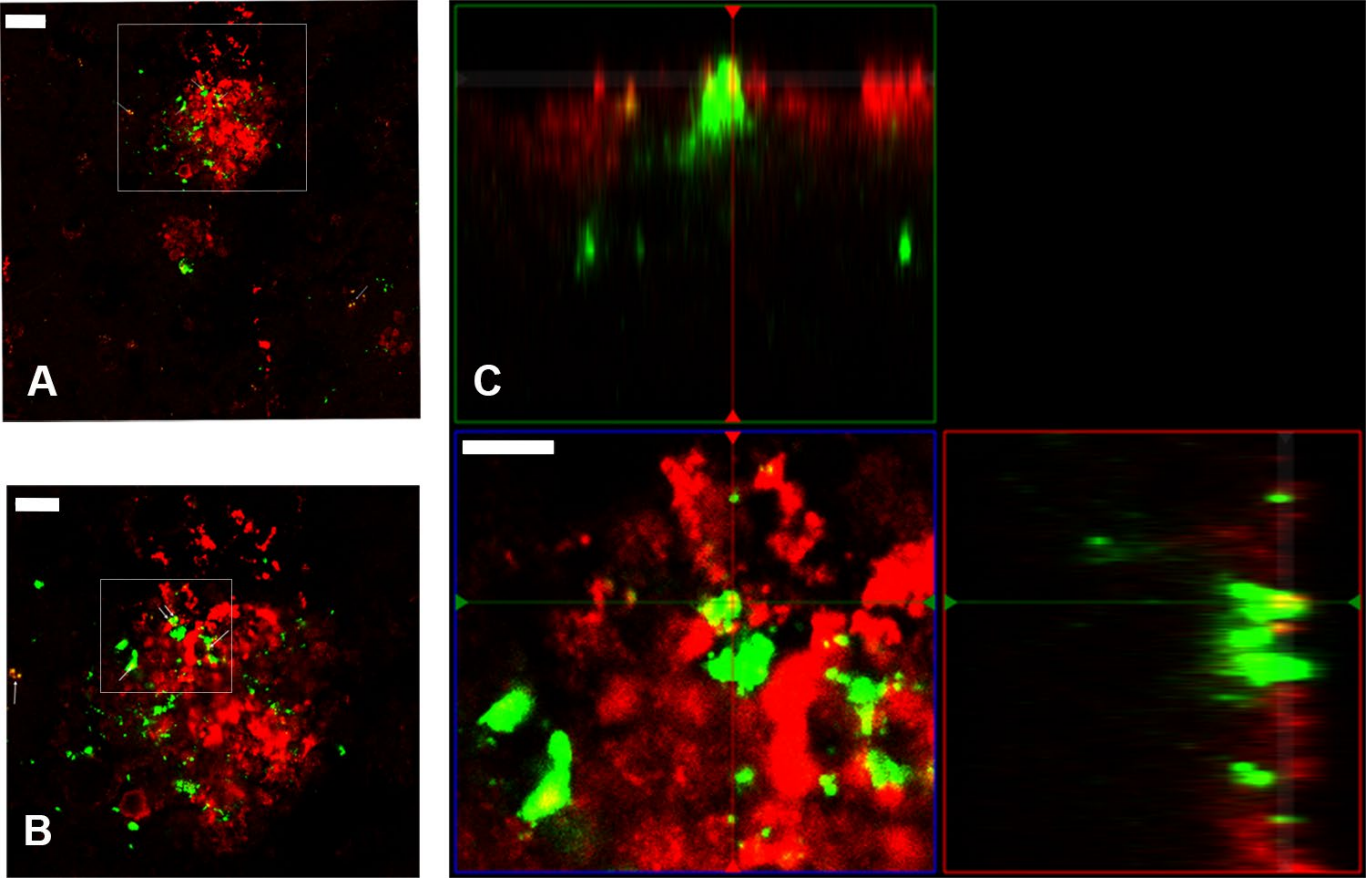
Aβ is located in microglial lysosomes. (**A**) TgSwe brain section showing an amyloid plaque surrounded by microglia with double staining using antibodies ab338 (red) and microglia by lysosomal marker CD68 (green), and (**B**) depicted with increased digital zoom. (**C**) Ortho image illustrating CD68- and ab338-staining being colocalized (yellow spots). The scale bars measure 20 µM, 10 µM and 5 µM and the gamma is set at 1.38/1.50, 1.42/1.58 and 1.42/1.42 (green/red channels) in (**A**–**C**) respectively. The images were obtained with a confocal microscope.

Phagocytosis is one of several cell uptake mechanisms. Cells of monocyte lineage, like microglia, highly express CD68 which localizes to endosomes and lysosomes^43^ and can serve as a microglial phagocytosis marker^52^ (Supplementary Fig. S4). To add evidence of microglial Aβ-uptake by phagocytosis while concomitantly further characterizing ab338 Aβ-labelling, brain sections of another APP-transgenic model, tgSwe, were stained with ab338 with and without a CD68-antibody. We found spots of co-localization of ab338 and CD68 suggesting Aβ-uptake by phagocytosis in tgSwe mouse brain (Fig. 4A–C). Tissue treatment with fluorophore-conjugated secondary antibodies alone showed that the signal was due to binding of primary antibodies (data not shown).

### Aβ-immunolabelling in microglia with transmission electron microscopy

**Figure 5:**
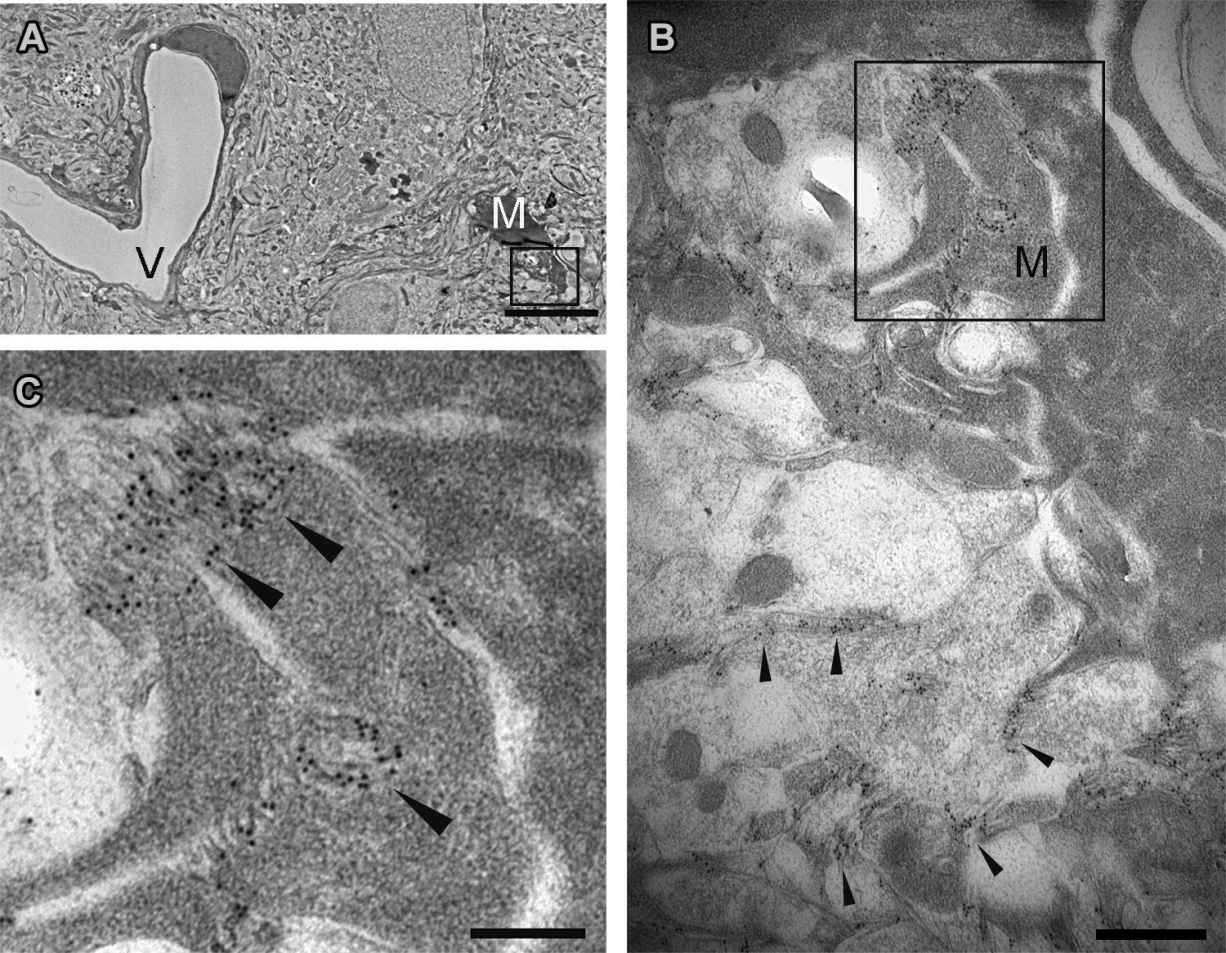
Ultrastructural illustration of a microglia located close to Aβ-immunoreactive fibrillar material in tgArcSwe mouse brain. (**A**) Transmission electron microscopy of tgArcSwe mouse brain showing a microglia (M) and a blood vessel (V). (**B**) At higher magnification, the ultrastructural microglial morphology of the microglial cell is appearent with Aβ-fibrils visualized by ab338 immuno-gold labelling in the rim of and in the microglia. (**C**) Focusing on the microglia that are attracted towards the amyloid fibrils and ab388 also label within the microglia suggesting Aβ-uptake. For structural information, fibrillar labelling with ab388 is illustrated by arrows (**B**,**C**). The scale bars measure 5 µm (**A**), 0.5 µm (**B**) and 0.2 µm (**C**).

Further examining ab338 labelling and microglial Aβ-uptake in situ in tgArcSwe mice at the ultrastructural level, ab338-immunogold particles labelled Aβ-fibrillar structures. The microglial processes were directed toward ab338-labelled Aβ-fibrils seemingly enconvuluting them. Intriguingly ab338-labelling was also present in the cell soma, indicating Aβ internalization by microglia in tgArcSwe mice (Fig. 5A–C). The distinct microglial anatomical structure was recognizable at the ultrastructural level, and confirmed by Iba-1 labelling of a microglia infiltrating an amyloid plaque (Supplementary Fig. S5).

## Discussion

APP-metabolites and Aβ-peptides are challenging to assay in tissues and body fluids due to their relatively low abundance, aggregation propensity and the complexity with many types of Aβ-species in tissues. In the current study, we characterized a novel antibody raised against the Aβ-mid-region Aβ_21–34_. The binding site was identified by biochemical and computational methods. The affinity-purified polyclonal antibody ab338 showed preferential binding to Aβ-peptides with C-terminal aa 34 (e.g. Aβ_1–34_). Such Aβ_x–34_ fragments could be detected by competitive ELISA at pM-concentrations. In the indirect ELISA, the only peptide with relevant binding except the Aβ_21–34_ peptide was Aβ_15–28_. This was consistent with ab338 binding to spot-synthesized peptides having a high β-turn score. Mapping the Aβ-epitope of ab338 by ELISAs and spot-synthesized Aβ-peptides gave overall consistent results with some discrepancies. This may be due to the spot-synthesized peptides presumably being more accessible for antibody binding as each spot had much more peptide compared to the indirect ELISA, although this in part was accounted for by using a lower ab338 concentration. As according to the analyzes of membrane spot-synthesized consecutive Aβ-peptide sequences, ab338 binding to multiple staggered sequences was observed and the signal did not very much depend on a specific aa, as the antibody bound from Aβ_19–28_ until Aβ_25–34_. We therefore asked if ab338 could be detecting a folding structure. Aβ-monomers aggregate into fibrils with a β-sheet structure composed of β-strands segments of Aβ wherein the peptide backbones are connected as the peptide flip over in a β-turn region (loop or reverse turn)^51^. The aggregation propensity of Aβ-peptides can be calculated by the Tango software, which has shown good prediction of peptide folding structure and aggregation-properties^53^. Calculating the β-turn conformational score with Tango aligned well with the findings of in vitro epitope-mapping suggesting that ab338 recognized a β-turn structure in Aβ. Preference of ab338 towards the β-turn in Aβ was supported by a negative outcome when instead trying to align α-helix and β-strand scores of the spot-synthesized Aβ-peptides with binding data. The Tango β-turn data also aligned well with structural analysis of Aβ_1–40_ fibrils with aa12-24 and aa30-40 forming β-sheets separated by a turning sequence at aa 25–29^54^. Thus the ab338 antibody seemed to bind Aβ in the β-turn region, preferentially β-turn and a C-terminal aa34. Still, given the high prevalence of full-length Aβ in brains of aged APP-transgenic mouse, we do not exclude that immunofluorescent staining to some extent is due to ab338 also recognizing longer Aβ-peptides that contain the Aβ_21–34_ domain e.g. Aβ_1–40_ and Aβ_1–42_ or other Aβ-species harboring this domain.

Existing Aβ antibodies mostly recognize the Aβ terminals either the N-terminal fragment of Aβ e.g. 6E10^55^ or a C-terminal epitope. Such antibodies are commonly combined to create sandwich Aβ-ELISAs to determine full-length peptides e.g. Aβx-42^56^. In contrast the 4G8 antibody recognize an epitope close to the α-cleavage site in the Aβ-domain^57^. Still there are few antibodies targeting the Aβ-mid-domain sequences and which thereby enable specific detection of mid-domain peptides. Such Aβ-mid-domain fragments may be released directly from APP or result from enzymatic cleavage of full-length Aβ-peptides^17,25^. Alternate APP-processing include a second BACE1 cleavage suceeding the initial BACE1/γ-secretase cleavage generating Aβ_1–34_ peptides^58,59^. Consistent with such cleavage the Aβ_1–34_ level in CSF is reduced following BACE-1 inhibiton and γ-secretase modulation^60,61^. Developing AD therapeutics like β-/γ-secretase inhibitiors is challenging^62,63^ and assaying a biomarker like CSF Aβ_1–34_ might aid in adjusting dosage and monitoring target engagement. ELISAs with Aβ C-terminal aa34 specificity would be advantageous compared to mass spectrometry to quantify such Aβ-species for logistical reasons. Aβ-mid-domain fragments could be a useful measure of Aβ-degradation by enzymatic breakdown, with neprilysin as the most well established degrading enzyme^64^. Neprilysin typically cuts the Aβ-peptides at the N-and C-terminals but cleavage sites also include the Aβ-mid-region at aa 19–20 and 33–34^65^. Additional enzymes may catabolize Aβ-peptides in the extracellular space. Examples are angiotensin converting enzyme (ACE)^66^ and matrix metalloproteinases (MMPs)^67^ with cleavage sites at aa 20/21, aa 33/34 and 34/35^65^ generating Aβ_21–x_ and Aβ_x–34_ peptides. In the intracellular compartments other enzymes like IDE, Cathepsin D and pre-sequence protease (PreP) complement the extracellular catabolic enzymes of which the latter two are suggested to generate Aβ_21–34_ and Aβ_x–34_ peptides^68,69^. Despite their low aggregation potential, reports indicate retained neurotoxicity of these shorter mid-domain peptides and the need for endolysosomal degradation and detoxification^51,69–71^.

Glial uptake of Aβ-peptides from the extracellular space, has until recent years been controversial in vivo^37^ and mainly supported by experimental in vitro data. The ab338 antibody displayed good immunolabelling, presumbaly due to preferential binding to a strech of amino acids forming a β-turn in Aβ. This region is likely more accessible for antibody binding since the peptides are interlinked in the β-strand regions. We have previously described astroglial responses to brain amyloid pathology^72^. Here we show ab338 Aβ-immunstaining located to microglia in situ at the ultrastructural level, with amyloid fibrills seemingly engulfed by microglia. Confocal microscopy with z-stacking at the light microscopic level was consistent with transmission electron microscopy data and added evidence of in vivo microglial Aβ-phagocytosis. Plaque formation attracts microglia and these plaque-attractated microglia aquire a disease associated microglial (DAM) phenotype^45^ also refered to as microglial neurodegenerative (MnDG) phenotype^73^. The DAM microglial phenotype is among others characterized by upregulation of *Axl* and *TREM2*^74^. These are phagocytic microglial receptors upregulated with neurodegeneration^75^ including in AD in the vicinity of senile plaques^44^. Both receptors constitute potential therapeutic targets and their role in Aβ-amyloid phagocytosis deserves further attention.

## Conclusions

Here we describe a novel Aβ mid-domain antibody, ab338 that preferentially binds a β-turn region in Aβ-peptides and a C-terminal aa 34. By ultrastructural- and light-microscopy histological techniques, we demonstrate microglial Aβ-uptake in situ supporting the more recently acknowledged role of microglia to Aβ-phagocytosis. Aβ-mid-domain antibodies like ab338 may complement existing laboratory tools when investigating APP processing and Aβ-pathology in biological samples and may be useful for theragnostic purposes.

## Methods

### Generation of polyclonal antibodies

The ab338 rabbit antibody was raised against a human Aβ21–34-peptide which had been conjugated to keyhole limpet hemocyanin (KLH) with maleimide reagent via its N-terminal cysteine (KLH-Cys-Aβ21–34; NH_2_-KLH-C-AEDVGSNKGAIIGL-COOH). The rabbit was immunized with a 1:1 (v/v) mixture of immunogen (100–200 µg) and Freund’s complete adjuvant and vaccinated three times with Freund’s Incomplete Adjuvant. The serum was purified by affinity chomatography against the Aβ_21–34_-peptide immobilized on an antigen-coupled sepharose column that subsequently was washed and eluted with 0.1 M glycine–HCl (pH 2.5) followed by rapid neutralization. This was all done at Agrisera (Agrisera, Vännäs, Sweden, ethical permit id A146–12).

### Indirect Aβ ELISA

MaxiSorp plates (Nunc, ThermoFischer, Waltham, MA, USA) were coated overnight (o.n.) with 0.9 pmol/well of the immunizing antigen, Aβ_21–34_, another Aβ-peptide, scrambled Aβ peptide or vehicle alone overnight (o.n.) at 4 °C. The following peptides were used (Aβ_15–28_, Aβ_16–24_, Aβ_18–26_, Aβ_21–31_, Aβ_21–34_, Aβ_21–35_, Aβ_35–40_, Aβ_37–42_, Innovagen, Lund, Sweden, Aβ_21–35_; Eurogentec, Belgium, Aβ_1–42scrambled_ #A-1004-1, rPeptide, Watkinsville, GA, USA). Most peptides used for coating harbored an N-terminal cysteine with an Ahx-spacer. The peptides were all dissolved in dimethyl sulfoxide (DMSO) and diluted in phosphate buffered saline (PBS; 137 mM NaCl, 2.7 mM KCl, 10 mM Na_2_HPO_4_, 2 mM KH_2_PO_4_, pH7.4). Next day, the wells were blocked with 1% (w/v) bovine serum albumin (BSA) in PBS for 1 h at 37 °C. The blocking solution was replaced by ab338 (0.5 µg/ml) and the plates incubated for 30 min at room temperature (RT). Next, the wells were incubated with a horseradish peroxidase (HRP)-conjugated secondary goat anti-rabbit antibody (0.125 µg/ml; #P0448; Dako, Glostrup, Denmark) for 30 min at RT. Both antibodies were dissolved in 0.1% BSA in PBS. All incubations were done with rotation, and between each step the fluid was aspirated and the plates washed three times with PBS with 0.1% (v/v) Tween-20 (PBS-T) except after the blocking. The ELISA plates were developed with K-Blue TMB Substrate (#331177; ANL-produkter, Sweden) at RT for 5 min, and the reaction was stopped with an equal volume of 0.4 M H_2_SO_4_. The plates were read at 450 nm in a SpectraMax 190 spectrophotometer and the results were analyzed with SoftMax Pro software (Molecular Devices, Palo Alto, CA, USA).

For estimation of the average affinity of the ab338 antibody an indirect ELISA was performed as described as above with some adjustments. The MaxiSorp plates were coated with KLH-Cys-Aβ_21–34_ (1 ng/well; Innovagen, Lund, Sweden) diluted in PBS o.n. at 4 °C. After blocking plates, the wells were incubated with ab338 at increasing concentrations (6.67 × 10^–12^–6.67 × 10^–8^ M; 0.001–10 µg/ml). The plates were then incubated with the HRP-conjugated anti-rabbit antibody (0.125 µg/ml, as described above), and finally development with K-Blue TMB Substrate as described above. The plates were read at 640 nm by a spectrophotometer as previously described.

### Competition Aβ ELISA

MaxiSorp plates were coated with KLH-Cys-Aβ_21–34_ (1 ng/well) in PBS o.n. at 4 °C. Next day, they were blocked with 1% (w/v) BSA in PBS for 3 h at RT. Meanwhile the competing Aβ-peptides (Aβ_1–34_, Aβ_21–34_, Aβ_21–31_, Aβ_21–35_, Aβ_1–40,_ Aβ_1–42_, the latter two from American Peptide Company, Sunnyvale, CA, USA) were at increasing concentrations (5 × 10^–11^–5 × 10^−5^ M) allowed to incubate for 2 h with the ab338 antibody (3 nM) in 0.1% BSA in PBS in wells of a non-binding plate (#655901-GBO; Greiner Bio-One, Frickenhausen, Germany). The blocking solution was aspirated and the ab338-Aβ-peptide mixture transferred to MaxiSorp plates for a 15 min incubation. The samples were then replaced by a secondary HRP-conjugated anti-rabbit antibody (0.125 µg/ml, described above) for a 1 h incubation. In each step, the plates were allowed to rotate and between each step, except after the blocking, the fluid was aspirated and the plates washed three times with PBS-T. All incubations were performed at RT unless otherwise specified. The ELISA was developed by incubation with K-Blue Substrate TMB (described above) and the reaction stopped by H_2_SO_4_ (0.4 M). The plates were read and analyzed with a spectrophotometer (450 nm) as described for the indirect ELISA.

### Epitope mapping by spot-synthetized peptides

16 Aβ-peptides, each 10 aa long with consecutive sequences starting at Aβ_13–22_ and ending with Aβ_28–37_ were directly synthezised covalently to a cellulose-ß-alanine-membrane with a N-terminal acetyl (JPT Peptide Technologies, Berlin, Germany). The membrane was soaked in methanol prior to incubation with ab338 (0.1 µg/ml). Subsequently, the membrane was incubated with HRP-conjugated goat anti-rabbit antibody (0.16 µg/ml, #31460 Pierce, Thermo Fischer, Waltham, MA, USA) and developed with LumiGLO (#547100, KPL, Kirkegaard & Perry Laboratories (KPL), Gaithersburg, USA). Between each step, the membrane was washed three times in Tris buffered saline (100 mM Tris, 150 mM NaCl) with 0.1% Tween-20 but substituted with LumiGLO washing solution in the last two washings prior to applying the LumiGLO substrate solution. The optical density of the immunoreactive signals was determined by using the ImageJ software (National Institute of Mental Health, Bethesda, MA, USA).

### Peptide aggregation and β-turn

Protein folding of peptides, including total β-turn score, α-helical score and β-strand score of the various Aβ-peptides was calculated by the Tango software^53^.

### Transgenic mice

Transgenic mice overexpressing human APP harboring the Arctic (E693G) and the Swedish (K670M/N671L) double mutation (tgArcSwe) or the Swedish double mutation only (tgSwe) were used to investigate localization of Aβ to microglia or brain macrophages, hereafter referred to as microglia. Homozygous transgenic mouse expressing CX3CR1-GFP (stock #005582, Jackson Laboratory, Bar Harbor, Maine, USA)^76^, was bred with hemizygous tgSwe to generate double transgenic tgSwe x CX3CR1-GFP mouse. Use and handling of animals were approved by the Biological Research Ethics Committee in Norway (Norwegian Animal Research Authority (NARA) permits id: 5693, 6006 and 7240.

The mice were fed ad libitum and housed under standard conditions with a 12 h light/dark cycle. DNA from ear cartilage was used to detect the transgene by polymerase chain reaction (PCR) as previously described^77^. TgArcSwe- and tgSwe mice were sacrificed at the age of 12 and 18 months respectively for investigation of microglial Aβ-localization. Briefly the mice were given a ZRF-cocktail (zolazepam; 18.7 mg/ml, tiletamine; 18.7 mg/ml xylazine; 0.45 mg/ml; fentanyl; 2.6 µg/ml, 0.75 ml/g body weight) or a mixture of ketamine (300 mg/kg body weight) and medetomidine (4 mg/kg body weight) for anesthesia. When the mice lacked pain reflexes they were transcardially perfused, decapitated and the brains quickly dissected. For light microscopic analyses the mice were perfused with 0.9% (w/v) saline and the brains fixed in 4% paraformaldehyde (PFA) in Sørenson’s phosphate buffer (SPB; 23 mM KH_2_PO_4_, 70 mM Na_2_HPO_4_*2H_2_0, 5 mM NaN_3_, pH7.4) o.n. at 4 °C. For ultrastructural analyses, the mice were perfused with a mixture of 4% PFA and 0.1% glutaraldehyde in 0.1 M phosphate buffer for 15 min subsequent to a flush with 2% dextran sulfate in phosphate buffer.

### Microscopy and immunostaining

#### Immunohistochemistry at the light microscopic level

For immunofluorescence, tgArcSwe brains were embedded in paraffin and coronal sections (6 µm) prepared. Prior to immunostaining, the sections were deparaffinized by serial immersion in xylene, 96% ethanol, 70% ethanol and finally water. All steps were repeated twice for 3 min. Immunostaining was performed as previously described^77^ with modifications. For Aβ- and microglial visualization in tgArcSwe brain, sections were immersed in PBS prior antigen retrieval by microwave treatment in citrate buffer (25 mM) and 70% (v/v) formic acid (5 min). The tissue was made permeable by immersion in 0.4% Triton X-100 in PBS (v/v) for 5 min. Sections were blocked with Dako protein block (#X0909, Dako, Glostrup, Denmark) and thereafter incubated with the primary antibody (0.5 µg/ml ab338, rabbit polyclonal) in PBS-T o.n. at 4 °C. In negative control experiments, PBS-T alone was used. Next day, the sections were rinsed in PBS prior to incubation for 30 min at RT in the dark with goat anti-rabbit antibody conjugated to Alexa-Fluor 488 (2 µg/ml, #A-11034, Thermo Fischer, Waltham, USA) alone or in combination with DyLight 594 labeled Lycopersicon Esculentum Tomato Lectin (2 µg/ml, #DL-1177, Vector laboratories, Burlingame, CA, USA) in PBS-T. The sections were mounted in SlowFade Gold Antifade Reagent with DAPI (#S36942 Molecular Probes, ThermoFischer, Waltham,USA) and sealed by use of nail polish.

TgSwe and tgSwe x CX3CR1-GFP brains were cryoprotected by serial immersion in 10%, 20% and finally 30% (w/v) sucrose in 0.1xSPB, each step o.n. at 4 °C. Coronal section (20 µm) were cut with a sledge microtome and stored in 10 mM sodium azide in 0.1xSPB. For immunofluorescence staining with ab338 and CD68 (rat-monoclonal antibody, #137001, BioLegend, San Diego, CA, USA) sections were mounted on SuperFrost Plus slides (#631-9483, VWR) and treated as described above, but antigen retrieval was omitted. Following o.n. incubation with CD68-antibody (1 µg/ml) alone or together with antibody ab338 (0.5 µg/ml), sections were rinsed in PBS and incubated with appropriate secondary antibodies labeled with Alexa fluorophores at 2 µg/ml (goat anti-rabbit antibody coupled to Alexa Fluor 594, #A-11037 and donkey anti-rat antibody coupled to Alexa Fluor 488, #A-21208, Thermo Fischer, Waltham, USA). When staining tgSwe × CX3CR1-GFP brain with CD68-antibody, donkey anti-rat antibody coupled to Alexa Fluor 594 was used at 2 µg/ml (#A-21209, Thermo Fischer, Waltham, USA). Finally sections were mounted in ProLong Diamond Antifade reagent with DAPI (#P36966, Molecular Probes, ThermoFischer, Waltham, USA) or counterstained with 1 µg/ml Hoecht33432 (#BTIU40047, VWR).

Confocal images were obtained using a LSM 510 Meta confocal microscope (Zeiss) and 40 × or 63 × oil immersion objectives. Z-stacking were done by use of the 63 × objective at 0.4 µm and 1.0 µM intervals for tgArcSwe and tgSwe brain sections respectively. The fluorophores were excited at 405 nm, 488 nm and 561 nm wavelengths at equal pinhole size. Detection of the DAPI, Alexa Fluor 488, Alexa Fluor 594 and Dylight 594 fluorophores were done sequentially and images merged as to outline fluorophore localization. A Carl Zeiss inverted microscope (Axio Observer Z1) equipped with a Hamamatsu ORCA Flash 4.0 camera were applied to obtain additional conventional fluorescence images. A 5 × fluar or a 40 × neofluar oil-immersion objective was used to obtain the images with DAPI, 38 HE Alexa and 63 HE Red Fluorescence filter cubes with set exposure times for the different filters of comparable sections. All subsequent image processing was performed in the ZEN Blue software (ZEN 2012, Carl Zeiss Microscopy, Germany).

#### Embedding and immunocytochemistry for electron microscopy

Pieces from mouse cerebral cortex (1.0 × 0.5 × 0.5 mm^3^) were dissected from 500 μm thick sections and embedded in Lowicryl HM 20 as previously described^72,78^. Cryoprotection and cryosubstitution were the two main steps of tissue preparation. Cryoprotection was undertaken by immersing tissues in phosphate buffered glucose, followed by increasing glycerol concentrations (10, 20, 30% (v/v)) before inserting the tissue specimens into liquid propane at − 190 °C in a liquid nitrogen cooled unit KF80 (Reichert, Vienna, Austria). Cryosubstitution was done in 0.5% uranyl acetate in anhydrous methanol at − 90 °C for 24 h in a cryosubstitution unit (AFS, Reichert). The temperature was gradually increased to − 45 °C and Lowicryl HM20 stepwise substituted by methanol. The specimens were polymerized under UV light for 48 h at − 45 °C.

Ultrathin sections (90 nm) were cut and transferred onto formvar-coated single hole grids and post-embedding immunogold labelling carried out. Briefly, the sections were incubated in 50 mM glycine in Tris buffered saline with 0.1% Trition X-100 (TBS-T) followed by 2% human serum albumin (HSA) in TBS-T (w/v). The primary antibodies diluted in 2% HSA TBS-T (ab338; 1:2000, Iba-1; 1:500) were applied to the sections for 2 h. The sections were rinsed twice with TBS-T before incubation with goat-anti-rabbit antibodies coupled to 15 nm gold particles in 2% HSA TBS-T for 1 h. For enhancing the contrast, uranyl acetate (Fluorochem) and lead citrate were used successively. The micrographs were obtained digitally by a transmission electron microscope (Technai 12, Hillsboro, Oregon, USA).

### Statistical analyses

The GraphPad Prism software (ver. 7.4, Graph Pad Software, La Jolla, USA) was applied for statistical analyses and to create graphs. Estimation of the equilibrium dissociation constant (K_D_) and the half inhibitory concentration (IC_50_) were done by the one site total function and curve fitting by adapting the results to sigmoidal dose–response function respectively.

### Use of experimental animals

(i) Use and handling of animals were approved by the Biological Research Ethics Committee in Norway (Norwegian Animal Research Authority (NARA) permits id: 5693, 6006 and 7240.
(ii) All experiments were performed in accordance with relevant guidelines and regulations.

## Supplementary information

Supplementary file1 (DOCX 4463 kb)

## Data Availability

The datasets used and/or analyzed during the current study are available from the corresponding author on reasonable request.
