# Supplementary material for: Analyzing microglial-associated Aβ in Alzheimer’s disease transgenic mice with a novel mid-domain Aβ-antibody
Source: Sci Rep. 2020 Jun 29;10:10590. doi: 10.1038/s41598-020-67419-2 (PMC7324359; doi:10.1038/s41598-020-67419-2)
Supplement: Supplementary file 1 — Supplementary file1 (DOCX 4463 kb) [file 41598_2020_67419_MOESM1_ESM.docx]

| **Suppplementary material**  Analyzing microglial-associated Aβ in Alzheimer’s disease transgenic mice with a novel mid-domain Aβ-antibody  **Kristi Henjum^1,2^, Vibeke Årskog^1^, Charlotte B. Jendresen^1^, Tormod Fladby^3^, Reidun Torp^4^ and Lars N.G. Nilsson^1*^**  **^1^**Department of Pharmacology, University of Oslo and Oslo University Hospital, Oslo, Norway  **^2^**Department of Geriatric Medicine, University of Oslo, Norway  **^3^**Department of Neurology, Faculty Division, Akershus University Hospital, University of Oslo, Lørenskog, Norway  **^4^**Department of Molecular Medicine, Institute of Basic Medical Sciences, University of Oslo, Oslo, Norway  *Corresponding author: Lars N.G. Nilsson, *lars.nilsson@medisin.uio.no*  Kristi Henjum:  ^1^Department of Pharmacology, University of Oslo and Oslo University Hospital, Oslo, P.O. 1057, Blindern, 0316 Oslo, Norway, and ^2^Department of Geriatric Medicine, University of Oslo, P.O. 4956, Nydalen, 0424 Oslo Norway. *kristi.henjum@medisin.uio.no*  Vibeke Årskog:  ^1^Department of Pharmacology, University of Oslo and Oslo University Hospital, Oslo, P.O. 1057, Blindern, 0316 Oslo, Norway. *vibeke.arskog@medisin.uio.no*  Charlotte B. Jendresen:  ^1^Department of Pharmacology, University of Oslo and Oslo University Hospital, Oslo, P.O. 1057, Blindern, 0316 Oslo, Norway. *cbjend@gmail.com*  Tormod Fladby:  **^3^**Department of Neurology, Faculty Division, Akershus University Hospital, University of Oslo, P.B. 1000, N-1478 Lørenskog, Norway. *tormod.fladby@medisin.uio.no*  Reidun Torp:  ^4^Department of Molecular Medicine, Institute of Basic Medical Sciences, University of Oslo, P.O. Oslo, Norway. *reidun.torp@medisin.uio.no*  Lars N.G. Nilsson:  ^1^Department of Pharmacology, University of Oslo and Oslo University Hospital, Oslo, P.O. 1057, Blindern, 0316 Oslo. *lars.nilsson@medisin.uio.no* Corresponding author.     \| **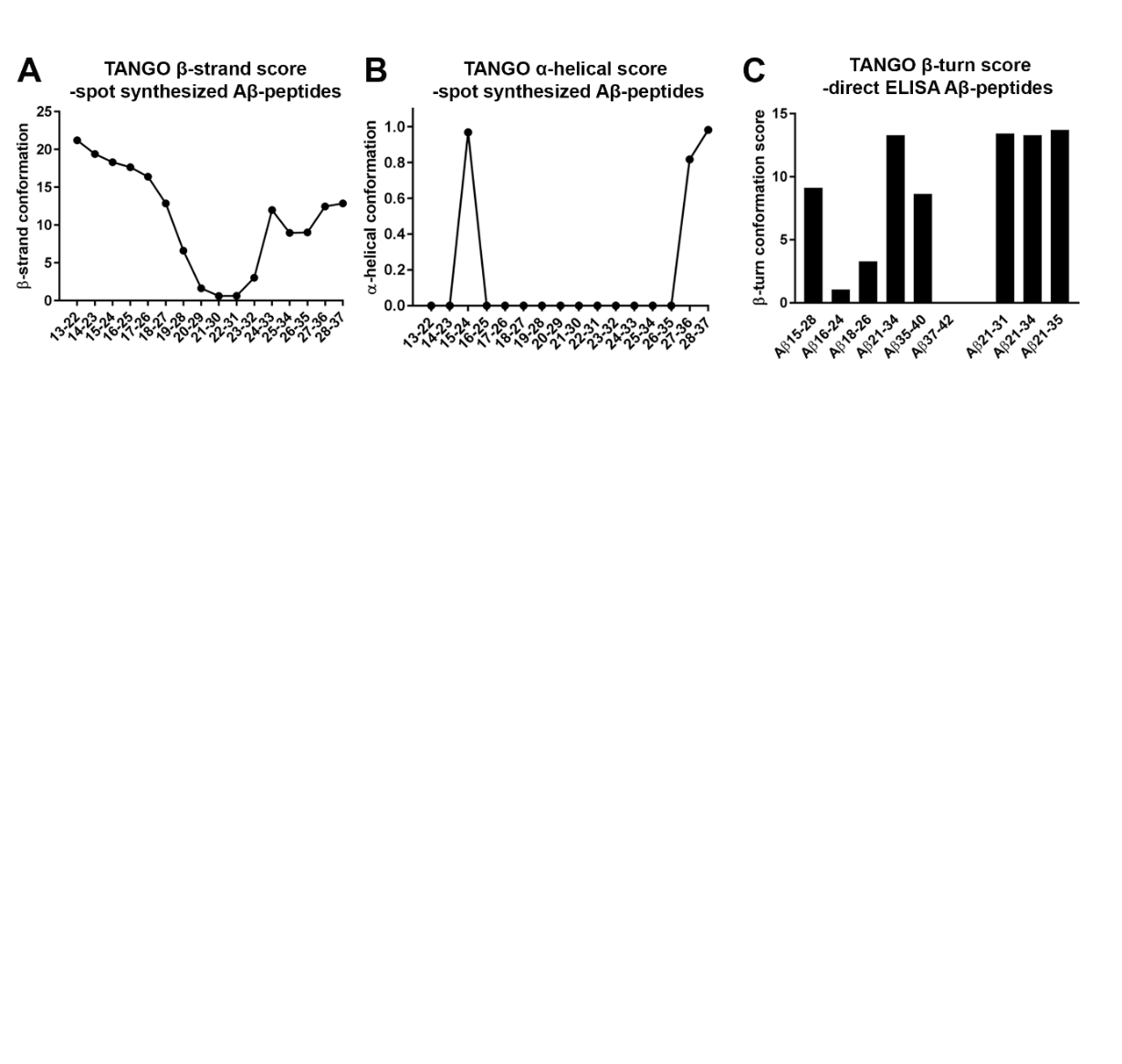** \| \| --- \| \| **Supplementary Figure S1:** Calculation by Tango software of (**A**) β-strand structure score of spot-synthesized Aβ-peptides (**B**) α-helical structure score of spot-synthesized Aβ-peptides. (**C**) Recognition of Aβ-peptides other than Aβ_21-34_ by antibody ab338 in solid phase binding assay (in Figure 1B) relates to sequence and β-turn Aβ structure score, as calculated with software Tango. \| | |
| --- | --- | --- | --- |
| \| **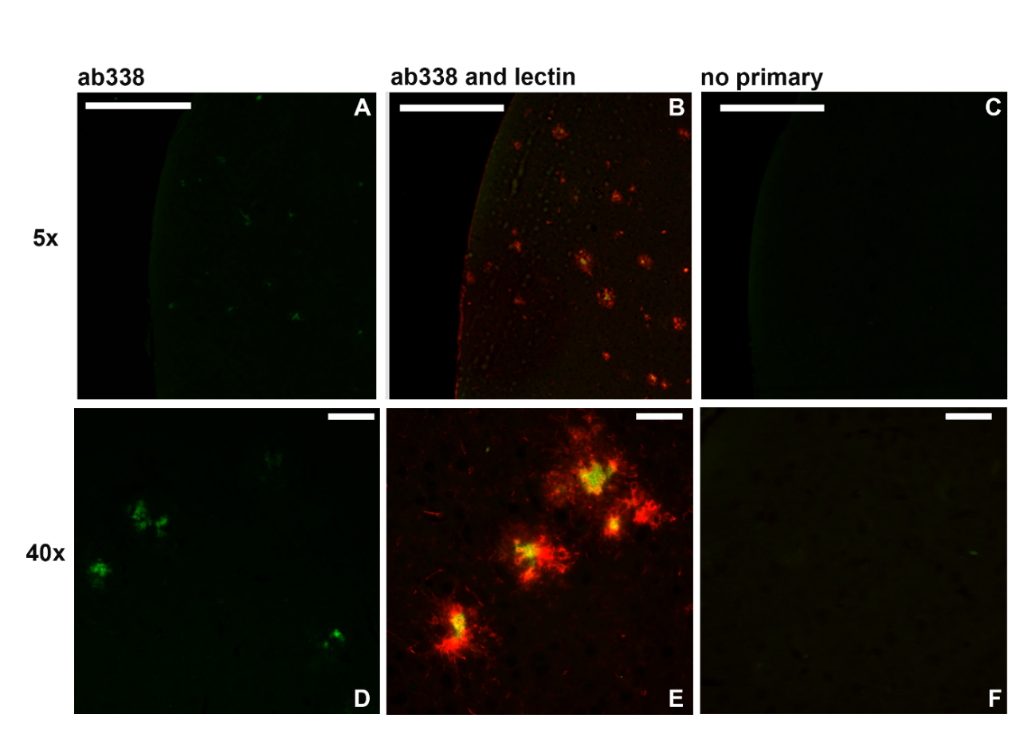** \| \| --- \| \| **Supplementary Figure S2:** Overview ab338 and tomato lectin staining of tgArcSwe mouse brain. The upper panels (**A-C**) present low magnifications (5x digitally zoomed ~2x, scale bars measure 500µm) while the lower panels (**D-F**) present higher magnification (40x, scale bars measure 50µm). Sections from tgArcSwe mouse brain stained with ab338 only (**A** and **D**). Co-labelling of antibody ab338 together with tomato lectin to visualize microglia in red (**B** and **E**)**.** Omitting the primary antibody ab338 results in faint background staining (**C** and **F**). All images are generated by merging images obtained in the green and red filters with equal adjustments, including gamma set at 1.7 and 1.45 at 5x and 40x magnification respectively, in the green filter and at 1.00 at both magnifications in the red filter. \|   **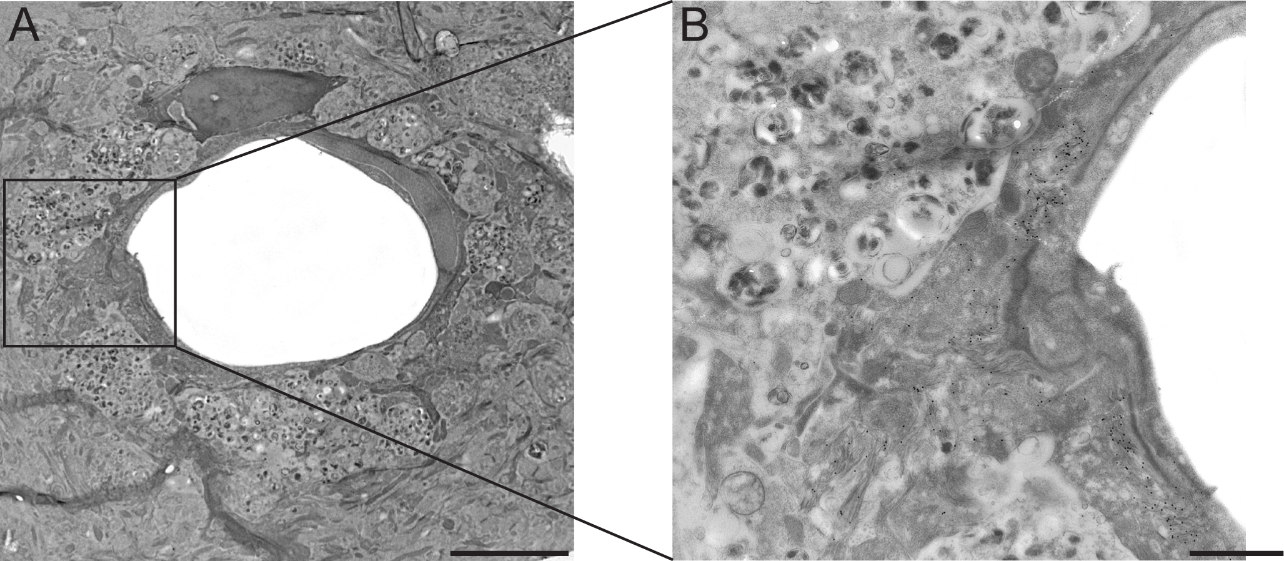**  **Supplementary Figure S3:** (**A**) An arteriole within layer III-IV of the primary cerebral cortex of tgArcSwe show accumulation of ab388-immunoreactivity close to the endothelial cell. The vessel is captured in an area close to a plaque, where the tissue show structural deterioration in terms of vesicles and thickening of the membrane. (**B**) Enlargement of the membrane to display the gold particles decorating the vessel wall. The scale bars measure 5µM (**A**) and 1µM (**B**).  **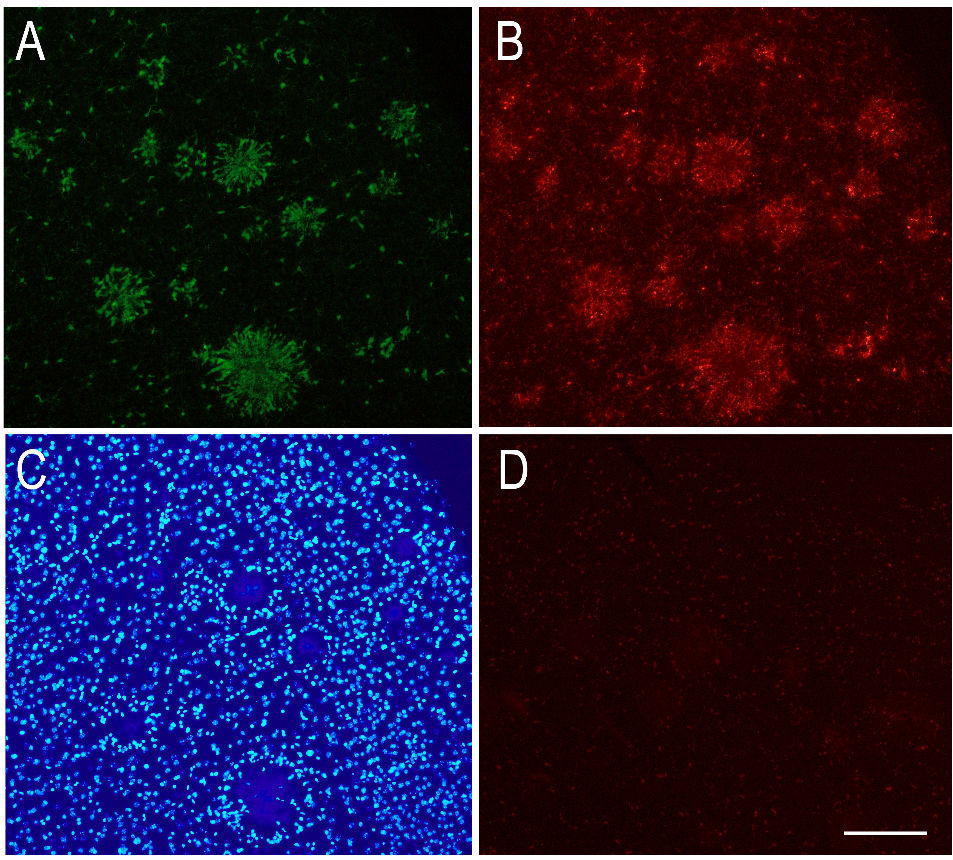**  **Supplementary Figure S4:** Brain tissue of a 19-month-old double transgenic tgSwe x CX3CR1-EGFP mouse. (**A**) Fluorescence emitted by microglial reporter CX3CR1-EGFP and (**B**) immunofluorescence when the tissue section is stained with primary CD68- and secondary Alexa 594 anti-rat antibodies. (**C**) Nuclear staining with Hoecht3342 to better show the location of amyloid plaques. (**D**) In a parallel negative control experiment, there is only faint background fluorescence when excluding the primary CD68-antibody. The scale bar measures 100µM (**A-D**). | |
| **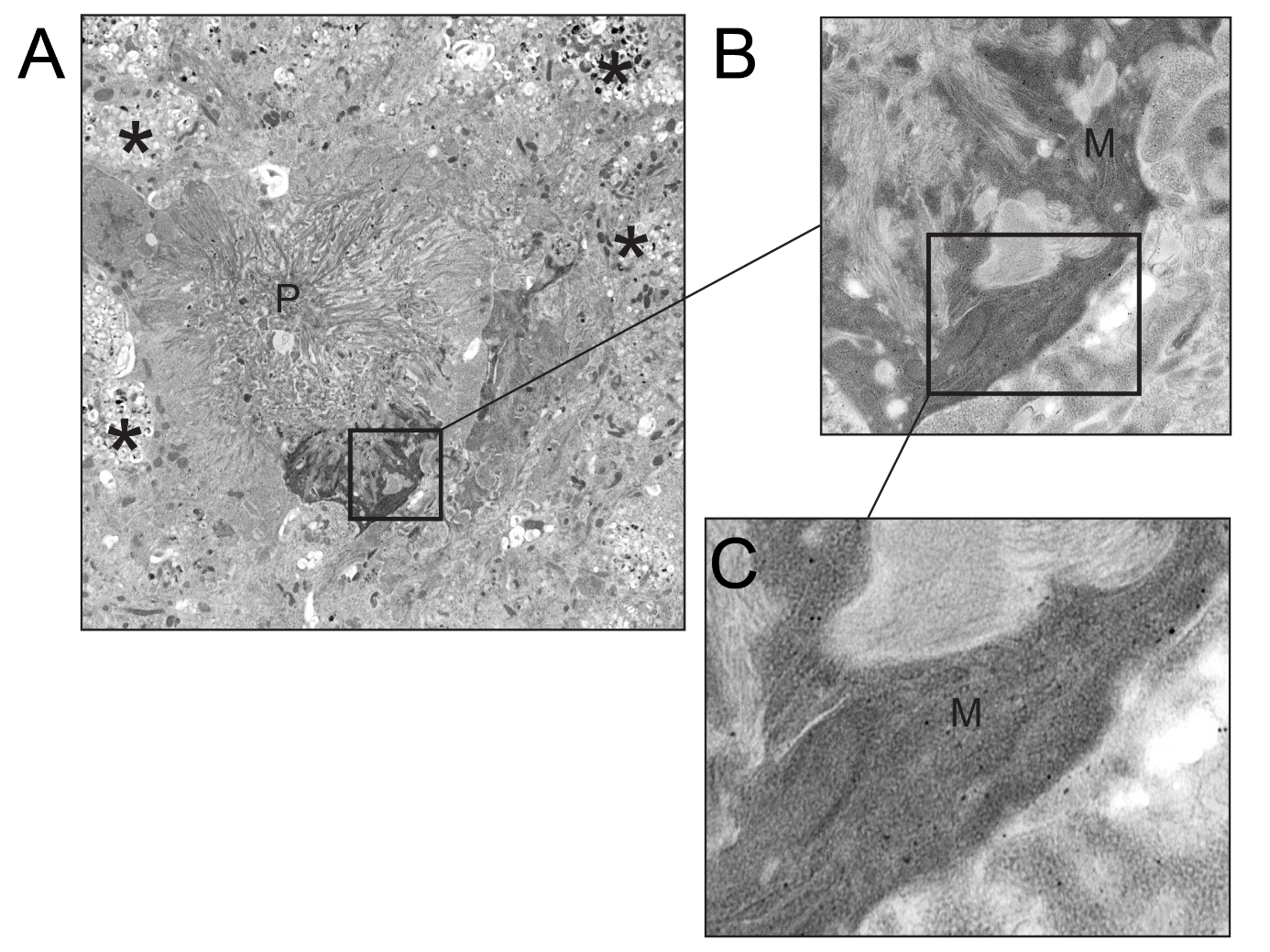** |  |
| **Supplementary Figure S5: Microglia at the ultrastructural level in tgArcSwe mouse brain**  **A**) An amyloid plaque (P) being infiltrated by a microglial cell as judged by morphology (4200x). **B**) At increased magnifications (16500x), Iba-1 immunogold-labelling is evident and verifies the recognition of microglia by structure at the ultrastructural level, **C**) and detail further magnified (2.5x). The asterisks indicate necrotic tissue. | |
|  | |
